# NEST3D printed bone-mimicking scaffolds: assessment of the effect of geometrical design on stiffness and angiogenic potential

**DOI:** 10.3389/fcell.2024.1353154

**Published:** 2024-03-07

**Authors:** Stephanie E. Doyle, Micaela Pannella, Carmine Onofrillo, Chiara Bellotti, Claudia Di Bella, Cathal D. O’Connell, Elena Pirogova, Enrico Lucarelli, Serena Duchi

**Affiliations:** ^1^ Electrical and Biomedical Engineering, School of Engineering. RMIT University, Melbourne, VIC, Australia; ^2^ Aikenhead Centre for Medical Discovery (ACMD), St Vincent’s Hospital Melbourne, Fitzroy, VIC, Australia; ^3^ Osteoncology, Bone and Soft Tissue Sarcomas and Innovative Therapies Unit, IRCCS Istituto Ortopedico Rizzoli, Bologna, Italy; ^4^ Department of Surgery, St Vincent’s Hospital, University of Melbourne, Fitzroy, VIC, Australia; ^5^ Department of Medicine, St Vincent’s Hospital Melbourne, Fitzroy, VIC, Australia

**Keywords:** negative printing, chorioallantoic membrane assay, angiogenesis, tissue engineering, bone regeneration

## Abstract

Tissue-engineered implants for bone regeneration require consideration regarding their mineralization and vascularization capacity. Different geometries, such as biomimetic designs and lattices, can influence the mechanical properties and the vascularization capacity of bone-mimicking implants. Negative Embodied Sacrificial Template 3D (NEST3D) printing is a versatile technique across a wide range of materials that enables the production of bone-mimicking scaffolds. In this study, different scaffold motifs (logpile, Voronoi, and trabecular bone) were fabricated via NEST3D printing in polycaprolactone to determine the effect of geometrical design on stiffness (10.44 ± 6.71, 12.61 ± 5.71, and 25.93 ± 4.16 MPa, respectively) and vascularization. The same designs, in a polycaprolactone scaffold only, or when combined with gelatin methacryloyl, were then assessed for their ability to allow the infiltration of blood vessels in a chick chorioallantoic membrane (CAM) assay, a cost-effective and time-efficient *in ovo* assay to assess vascularization. Our findings showed that gelatin methacrylolyl alone did not allow new chorioallantoic membrane tissue or blood vessels to infiltrate within its structure. However, polycaprolactone on its own or when combined with gelatin methacrylolyl allowed tissue and vessel infiltration in all scaffold designs. The trabecular bone design showed the greatest mineralized matrix production over the three designs tested. This reinforces our hypothesis that both biomaterial choice and scaffold motifs are crucial components for a bone-mimicking scaffold.

## Graphical Abstract

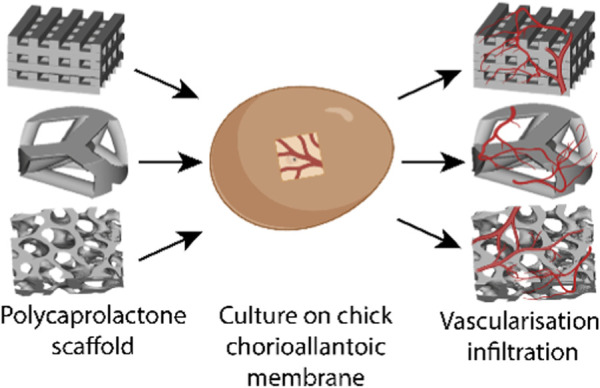

## 1 Introduction

Tissue-engineered scaffolds for bone regeneration are composed of the fabricated tissue, including mineralized tissue and a vasculature network, as well as the function of the tissue. For bone, a key function is structural—to support movement and respond to and transfer load (Gupta et al., 2006; Datta et al., 2008; Su et al., 2019). To address the mechanical properties of a bone regeneration scaffold, 3D printing offers a suitable solution to recapitulate the porosity and stiffness of native bone. However, the design of the scaffold is often limited to a basic logpile where straight lines are rotated by 90° for each subsequent layer with limitations in design complexities and porosities that can be achieved (Turnbull et al., 2018). Negative Embodied Sacrificial Template 3D (NEST3D) printing can be used to create highly intricate 3D geometries. In our previous work, we have demonstrated the capability of NEST3D printing to generate polycaprolactone (PCL) scaffolds with high design versatility and mechanical tunability (Doyle et al., 2021). With NEST3D, different motifs, such as biomimetic designs and lattices, can be designed to allow full interconnectivity and reach the relevant porosity of the bone (63.7%–91.4% for human trabecular bone) (Mullins et al., 2020; Porrelli et al., 2022; Stefanek et al., 2023). Biomimicry in porosity allows cell ingrowth and proliferation and diffusion of nutrients and oxygen, while high porosity and pore size have also been shown to favor both osteogenesis and vascularization (Sun et al., 2004; Mehdizadeh et al., 2015; Gupte et al., 2018; Wang et al., 2021; Sun et al., 2022). Although obtaining these properties is important, the design level of mechanical stability is still required (Zhao et al., 2002; Sun et al., 2004).

Bone contains a rich vasculature system which delivers oxygen and nutrients while removing waste from the cells (Jain et al., 2005; Lovett et al., 2009; Marenzana and Arnett, 2013; Ramasamy, 2017; Hendriks and Ramasamy, 2020). The vascular network of bone also provides access to a range of cells which can provide some self-regeneration potential to the bone (Lees and Partington, 2016; Khorshidi and Karkhaneh, 2018; Ansari et al., 2019). If the vasculature network is reduced or dysfunctional, then bone loss can occur (Marenzana and Arnett, 2013; Tomlinson and Silva, 2013). Therefore, it is important that the scaffold design promotes the growth of new vessels into the structure from the existing network, and so, a test to assess angiogenesis is needed (Risau, 1997; Vailhé et al., 2001).

While *in vitro* models to test angiogenesis continue to emerge, it is more common to see angiogenesis experiments conducted directly *in vivo* in small animal models including zebrafish, mice, rats, and rabbits (Dziubla and Lowman, 2004; Staton et al., 2004; Norrby, 2006; Staton et al., 2009; Song et al., 2014; Kim et al., 2020). With each animal, various angiogenesis assays can then be chosen based on the specific application including the corneal, dorsal air sac, chamber (ear and cranial), or subcutaneous implantation (Staton et al., 2004; Norrby, 2006; Staton et al., 2009). Regardless of the assay, for angiogenic applications, a structure is implanted in a vascularized area which then allows for vessel infiltration into the structure (dos Santos et al., 2019; Tamari et al., 2020). Along with imaging, the number, size, and location of vessels can be recorded to determine the degree of infiltration. However, as with all *in vivo* experimentation, there are several ethical and regulatory questions including potential pain and distress to animals, high costs, specialized surgical training for procedure, animal housing facilities, variable number of animals required for statistical significance, and laborious experimentation (Klaunberg and Lizak, 2004; Barré-Sinoussi and Montagutelli, 2015; Mukherjee et al., 2022).

**FIGURE 1:**
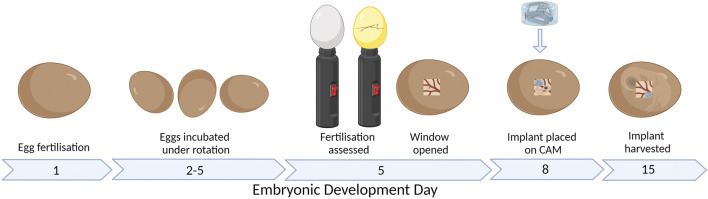
CAM assay workflow. Figure created with BioRender.com (29 January 2023).

An alternative assay is the chick chorioallantoic membrane (CAM) assay. The CAM is a highly vascularized membrane which begins developing between embryonic development days (EDDs) 3–5. It then rapidly grows, increasing from an area of 6 cm^2^ on EDD 6 to 65 cm^2^ by EDD 14 (Tanaka et al., 1986; DeFouw et al., 1989). Cell pellets, tissues, biomaterials, and bioscaffolds can be placed on the CAM and cultured to determine the pro-angiogenic nature of the samples (Figure 1). The implant can be analyzed by the visual assessment of the number of vessels growing into the implant and then harvested and sectioned to understand the development within the implant.

The chick embryo takes approximately 21 days to fully develop before hatching. The development of the nervous system begins from EDD 7, while a functional brain is developed by EDD 13. There is less consensus on when the perception of the pain exactly starts; what is known is that nociception starts after the second week (Kollmansperger et al., 2023; Weiss et al., 2023). Furthermore, the chick embryo is naturally confined within the shell until it hatches; therefore, it does not have any psychological discomfort from the isolation. Therefore, the CAM model and the zebrafish can be used within boundaries to ensure less discomfort and suffering compared to fully mature animals such as the mice, rats, or rabbits (Laschke et al., 2022). The implant is typically added to the CAM between EDD 6 and 10 (Figure 1) (Magnaudeix et al., 2016; Moreno-Jiménez et al., 2016; Moreno-Jiménez et al., 2018; Petrovova et al., 2019).

In our study, mechanical analysis was used to compare various designs and determine the values that correlate to the native bone tissue. Then, the CAM assay was used to test the vascularization potential of a NEST PCL scaffold, of varying designs, with or without soft gelatin methacryloyl (GelMA) hydrogel which has been photocrosslinked to produce the hydrogel scaffold (Doyle et al., 2021). Pure PCL and GelMA have been separately tested on the CAM model, with the literature showing little evidence to support vessel infiltration into the scaffolds (not just around); however, the combined materials have not been assessed (Cidonio et al., 2019; Aldemir Dikici et al., 2020; Kohli et al., 2020; Ren et al., 2021; Ibanez et al., 2022). Furthermore, PCL 3D printed structures of designs other than a basic 90° logpile have also not been assessed with the CAM model (Aldemir Dikici et al., 2020; Kohli et al., 2020; Ren et al., 2021). Throughout our study, we observed the effect of the scaffold design on the mechanical properties and infiltration of new vessels into the scaffolds. The evaluation of the scaffolds for their mechanical properties and vascularization infiltration takes one step forward in making a functional bone regeneration scaffold.

## 2 Materials and methods

### 2.1 Scaffold design and fabrication

Three designs were utilized during this study: logpile, Voronoi, and trabecular bone. All scaffold designs were of the same dimensions and were of 8 mm diameter and 5 mm height for mechanical testing, while scaffolds for the CAM experimentation were of 4 mm diameter and 2 mm height. The logpile was designed in SOLIDWORKS (Dassault Systèmes SolidWorks Corporation, Massachusetts, United States), the Voronoi in nTopology (nTopology, New York, United States) using the Voronoi lattice type, and the trabecular bone design was segmented from an MRI scan of human bone. The scaffolds were fabricated via the NEST3D printing protocol previously described (Doyle et al., 2021). In brief, the assembled sacrificial templates were printed with PVA (Formfutura, Nijmegen, Netherlands) using an UltiMaker S3 printer (UltiMaker, Utrecht, Netherlands). The templates were placed inside a custom-built chamber with medical grade PCL (PURASORB PC 12, Corbion Inc., Gorinchem, Netherlands) added on top. The system was heated to 70 °C, and 1 bar of air pressure was applied to allow the PCL to entirely fill the negative space of the template. The template was ultrasonicated (2800 Ultrasonic Cleaner, Branson Ultrasonics Corporation, Connecticut, United States) at 4°C–23 °C to rapidly dissolve the PVA template for 6–7 h to ensure the complete removal of all visible and non-visible traces of PVA (Doyle et al., 2021). The scaffolds were then sterilized using ethanol and UV light.

### 2.2 Mechanical testing

The compressive modulus of the scaffolds and human trabecular bone was assessed via compression testing at room temperature using a TA ElectroForce 5500 mechanical loading device (TA Instruments, Delaware, United States) fitted with a 50 lb load cell. The human trabecular bone was obtained from femoral condyles of donor patients undergoing total joint knee replacement for osteoarthritis (n = 2). The use of all human samples and procedures in this study was approved by the Human Research Ethics Committee Research Governance Unit of St. Vincent’s Hospital, Melbourne, Australia [HREC/16/SVHM/186], and all the experiments were performed in accordance with relevant guidelines and regulations. The physical dimensions of each sample (diameter, height, and mass) were taken prior to testing. The samples were compressed between two stainless steel plates in an unconfined setting. The bottom plate was in a fixed position, and the top plate moved following a ramp function at a rate of 0.01 mm s^–1^ until a total displacement of 30% of the sample height or until maximum machine capabilities (200 N). Load and displacement measurements were recorded and converted into stress (σ) and strain (ε) data using the measured cross-sectional area and its height. The stiffness was then computed using the slope of the stress–strain curve between a strain of 0.01 and 0.05. This range represented a linear region of the stress–strain curve and the maximum strain range that covered all groups. For each test group, a technical quadruplicate (n = 4) was used.

### 2.3 Generation of hydrogel scaffolds for the CAM

GelMA was synthesized and provided by TRICEP (Wollongong, NWS, Australia) and dissolved to the final concentrations of 60 mg/mL GelMA in sterile PBS (Sigma-Aldrich), containing 100 U/mL penicillin and 100 μg/mL of streptomycin (Gibco, Thermo Fisher Scientific Inc., Waltham, MA, United States). The concentration is reported in the paper as 6% GelMA. The photoinitiator lithium phenyl-2,4,6-trimethylbenzoylphosphinate (LAP) was obtained from Tokyo Chemical Industries (Tokyo, Japan), made up in stock 2% w/v solutions in PBS, and filter sterilized through 0.22-μm syringe filters. LAP was used in 6% GelMA at 0.06% w/v to induce photo-crosslinking. For the biphasic samples which included a NEST PCL scaffolds, the scaffold is first added to a PDMS mould of 4 mm diameter and 2 mm height. For all hydrogel-containing samples, 20 µL of GelMA hydrogel was added to the mold before UV irradiation as previously described, at room temperature for 60 s, using a 405 nm UV source (BioLambda, Sao Paulo, Brazil) with a light irradiance of 20 mW/cm^2^ (Onofrillo et al., 2021). All hydrogel scaffolds were transferred to an ultra-low attachment 96-well plate (Corning, Maine, United States) and washed with PBS 1x.

### 2.4 CAM model setup

The CAM model workflow is shown in Figure 1. Fertilized eggs were collected on EDD 1 or 2, cleaned with distilled water, and placed in a dedicated incubator at 37 °C and 56% humidity. Until EDD 5, the eggs were under gentle rotation to ensure the CAM does not attach to the outer shell. On EDD 4, the fertilized eggs were discriminated against unfertilized eggs by visualizing the presence of the blood vessel network using an LED lamp. On EDD 5, the eggs are wiped with 70% ethanol only where the window is cut; then, 2–3 mL of albumen is removed with a 21 G needle and syringe before the hole was closed with parafilm. A piece of clear tape was added vertically on the long side of the egg before a second small hole is made, and sharp scissors were used to cut the window of approximately 1 cm^2^. A stereomicroscope (SZO-T fitted with a C-P20 camera, OPTIKA Microscopes, Ponteranica, Italy) was used to assess the CAM and embryo to discriminate again between live embryos and unfertilized or deceased embryos. The window was then sealed with parafilm. For the remaining period, the eggs were incubated under static conditions. On EDD 8, the hydrogel scaffolds were placed on the CAM with 1–3 hydrogel scaffolds per egg. On EDD 12–15, the window was enlarged, and stereomicroscope images are taken before the hydrogel scaffolds are removed from the CAM. The hydrogel scaffolds were fixed with 1% paraformaldehyde (Sigma-Aldrich) for 2 h and stored in 30% w/v sucrose (Sigma-Aldrich) at 4 °C.

### 2.5 Cryo-embedding and sectioning

Hydrogel scaffolds were removed from sucrose, embedded in O.C.T. Compound (Tissue-Tek, Leiden, Netherlands) and flash frozen in liquid nitrogen. Cryosections of 7 μm thickness were cut along the axial plane. The cryosections were mounted onto Superfrost Plus adhesion glass slides (Thermo Fisher Scientific) for staining and imaging.

### 2.6 H&E staining

All steps were carried out at room temperature. Slides were brought to room temperature and then dipped in a fixative (50% ethanol, formalin, and glacial acetic acid in a ratio of 680:120:1) for 30 s. Then, they were dipped again in distilled water (dH_2_O) twice (fresh dH_2_O for each dip), immersed in Gill’s Hematoxylin for 45 s and dipped again in fresh dH_2_O. The slides were immersed in dH_2_O and ammonia (500:1) for 15 s, again dipped in fresh dH_2_O, and then, dipped in 80% ethanol. The slides were immersed in alcoholic eosin 0.5% for 10 s, then dehydrated and dipped in 95% ethanol twice (fresh ethanol for each dip), dipped in 100% ethanol twice (fresh ethanol for each dip), and cleared in xylene (Chem-Supply) 2 changes 30 s each. The slides were then mounted in the Eukitt mounting medium with glass coverslips on top.

The samples were imaged using an inverted Nikon Eclipse TE2000-U microscope (Nikon, Amsterdam, Netherlands) equipped with a DXM1200F camera, using ×4 and ×10 objectives, PhL and Ph1 filters (Nikon), and NIS-Elements software using Nikon objectives (Nikon).

### 2.7 Immunofluorescent staining

All steps were carried out at room temperature, unless otherwise stated, and in a humidity box. Slides were washed in PBS 1x (Euroclone, Milan, Italy) for 20 min, and then, hydrophobic borders were drawn around the sections using a liquid blocker pen (Vector Laboratories, Newark, United States) to limit the volume of reagent needed per slide. Blocking solution [0.3% Triton (Sigma-Aldrich)] in PBS, 1% bovine serum albumin (Sigma-Aldrich), and 1% serum were added on top of each section for 1 h. The blocking solution was removed, then the primary antibody, VE-Cadherin (rabbit, D87F2 XP, mAb#2500, Cell Signaling, Danvers, MA, United States), collagen type I (mouse, C2456, Sigma-Aldrich) (1:100 in blocking solution), or osteocalcin (mouse, 190125, R&D Systems, Minneapolis, United States) (1:50 in blocking solution) were added and incubated overnight at 4 °C. The next day, the sections were washed in PBS 1x, three times for 5 min each. The secondary antibody (Alexa Fluor^®^ 488 AffiniPure Goat Anti-Rabbit IgG, F(ab')₂ fragment specific, Jackson ImmunoResearch, West Grove, United States) or (Alexa Fluor™ 488 Rabbit anti-Mouse IgG, A11059, Life Technologies, Carlsbad, United States) was then added (1:200 in blocking solution) for 1 h. The sections were washed in PBS 1x, three times for 5 min each. Hoechst (H3569, Life Technologies) (1:500 in 0.3% Triton in PBS) was added to each section for 10 min before a final wash in PBS 1x, three times for 5 min each.

The samples were imaged using an inverted Nikon Eclipse TE2000-U microscope (Nikon, Amsterdam, Netherlands) equipped with a DXM1200F camera, a fluorescent lamp (Nikon), a Nikon Plan Fluor ×40 objective (Nikon), and NIS-Elements software (Nikon).

Fiji ImageJ software was used for imaging analysis and quantification. Specifically, the Bio-Formats Import option was used to transform the image and quantify the fluorescence signal. Duplicate images for each sample were used to quantify the signal emitted. The quantifications were performed by taking five randomly selected regions of interest (ROIs) per image.

Before choosing the areas of interest, the background signal was reduced to avoid false quantification values. Once the ROI was designed, the maximum, minimum, average value, and standard deviation of positivity were recorded. The area and the perimeter were evaluated using ROI Manager.

### 2.8 Alizarin Red S

Alizarin Red S staining (Alizarin Red S, Sigma-Aldrich, Burlington, MA, United States) was used to evaluate the degree of calcium mineralization of the tissues present inside the scaffolds. The cryosections were hydrated with H_2_O for 10 min and incubated with 2% Alizarin Red S solution for 10 min. The sections were then washed twice with H_2_O and mounted with ProLong™ Gold Antifade Mountant (Thermo Fisher, Burlington, MA, United States). The samples were imaged using an inverted Nikon Eclipse TE2000-U microscope (Nikon, Amsterdam, Netherlands) equipped with a DXM1200F camera, using Nikon Plan Fluor ×40 objective and A filter (Nikon), and NIS-Elements software using Nikon objectives (Nikon). Triplicate images for each sample were used to quantify the signal emitted. QuPath −0.4.3 software was used for quantification performed by selecting five random ROIs per image. Once the ROIs were chosen, the threshold of the positive signal for Alizarin Red was set. The total area and the positive area of the ROI were considered.

### 2.9 Statistics

For each experimental quantitative assessment, at least four replicates (n = 4) were used with data summarized as the mean with error bars representing standard deviation. All statistical analysis was performed using Prism 9 (GraphPad, San Diego, United States) with a statistical significance level ≤0.05. The normality of each dataset was first assessed using the Shapiro–Wilk test and passed. Then, significance was determined using a one-way ANOVA. In all graphs, stars represent the following: * is *p* ≤ 0.05; ** is *p* ≤ 0.01; *** is *p* ≤ 0.001; and **** is *p* ≤ 0.0001.

## 3 Results and discussion

### 3.1 Scaffold design and stiffness comparison

The design of a 3D printed scaffold can affect its subsequent mechanical properties. Three different scaffolds were fabricated with NEST3D to generate logpile, Voronoi, and trabecular bone geometries. The logpile design, consisting of straight lines with a 90° rotation in each subsequent layer, represents one of the most common 3D printing designs within the tissue engineering literature but has no physiological relevance. The Voronoi design is a mathematical model that can be applied both in 2D and 3D space and is said to resemble trabecular bone (Herath et al., 2021). Finally, the trabecular design represents the exact biomimetic condition as it is taken directly from a scan of human bone.

**FIGURE 2:**
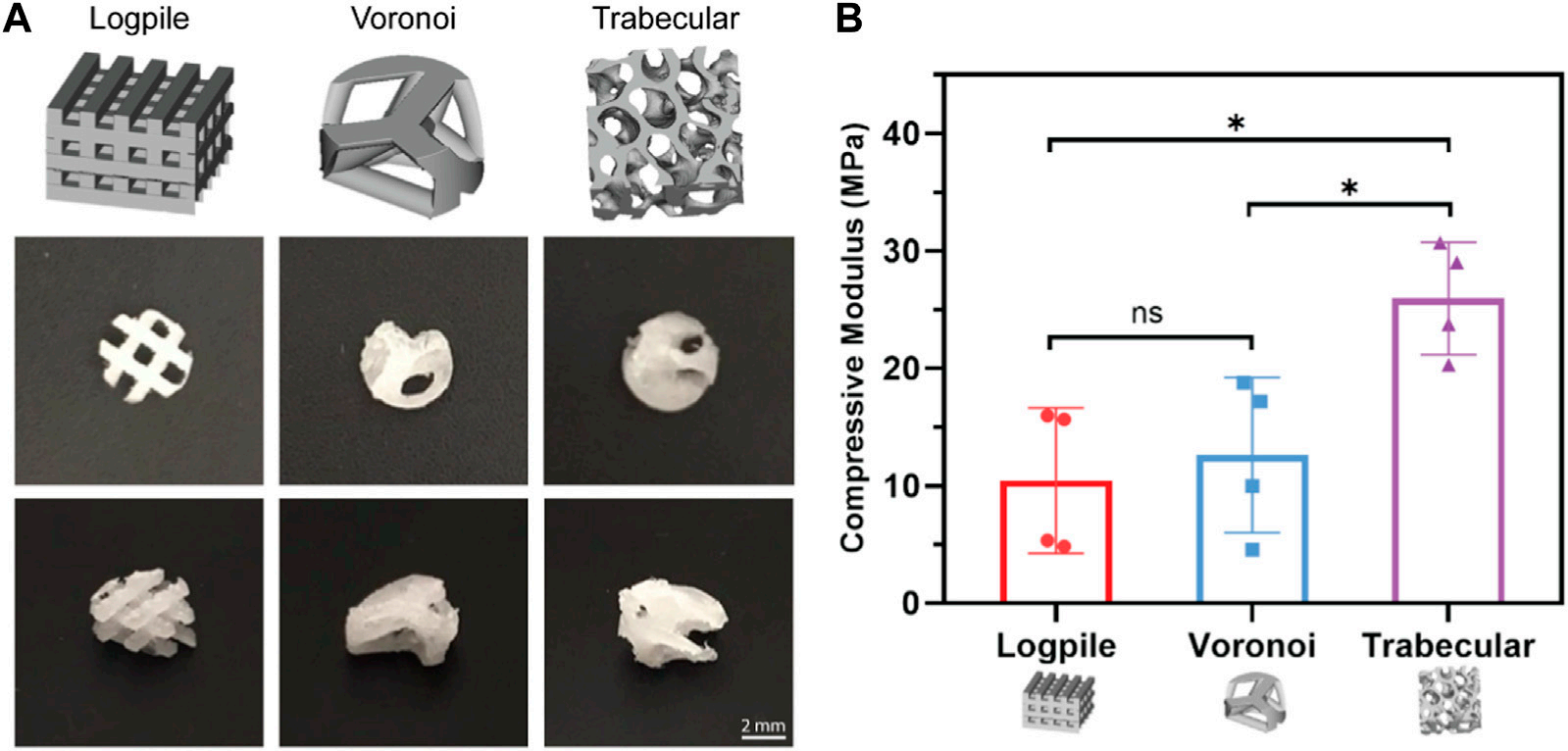
Scaffold designs and stiffness comparison. **(A)** CAD design of the three different geometrical designs (upper panel) and representative macroscopic images of each scaffold from different orientations (4 mm diameter, 2 mm height). **(B)** Bar graph represents the compressive modulus from unconfined compression of the NEST PCL scaffolds. All samples were of 8 mm diameter and 5 mm height. Error bars represent standard deviation, n = 4. * is *p* ≤ 0.05.

All three scaffold designs were fabricated from medical-grade PCL using the NEST3D technique (Figure 2A), and their stiffness was assessed with mechanical compression testing (Figure 2B). The trabecular design (25.93 ± 4.16 MPa) was significantly stiffer than the logpile and Voronoi (10.44 ± 6.71 and 12.61 ± 5.71 MPa, respectively).

### 3.2 CAM model setup: the weight test

To test the vascularization capacity of the three different designs, the CAM assay was used, providing valuable insights into their potential for fostering angiogenesis and tissue infiltration. The chorioallantoic membrane begins developing between EDD 3 and 5 and then rapidly increases in size from an area of 6 cm^2^ on EDD 6 to 65 cm^2^ by EDD 14 (Tanaka et al., 1986; DeFouw et al., 1989). This overall increase in the CAM surface area translates to an increased weight of implants that the CAM can withstand without damaging the native network. Only a limited number of papers relating to biomaterials on the CAM refer to the weight of the scaffold implanted, or the potential for a heavy scaffold to damage the CAM (Moreno-Jiménez et al., 2017). Of the very few papers that do report scaffold weight, Kanczler et al. (2007) had a mean weight of 29.6–30.0 ± 0.8–3 mg with no mention of this weight causing any damage to the blood vessels of the CAM (Kanczler et al., 2007). Not only a heavy scaffold can damage the CAM but also the entire scaffold can sink through the CAM (Magnaudeix et al., 2016). When this occurs, the scaffold may become embedded or engulfed in the yolk or amniotic sac with little to no interaction with vessels (Supplementary Figure S1). Therefore, when the scaffolds are not standing on top of the CAM when retrieved, the results cannot be considered reliable.

Previous experiences in our laboratory showed that an implant weight of approximately 80 mg over an area of approximately 64 mm^2^ caused damage to the vessels after only 2 hours when placed on the CAM on EDD 8. Therefore, in the preliminary experiments, we checked if the fully assembled hydrogel scaffolds (NEST PCL scaffold + GelMA, 4 mm diameter and 2 mm height) could be placed on the CAM (EDD 8) without damaging the network.

**FIGURE 3:**
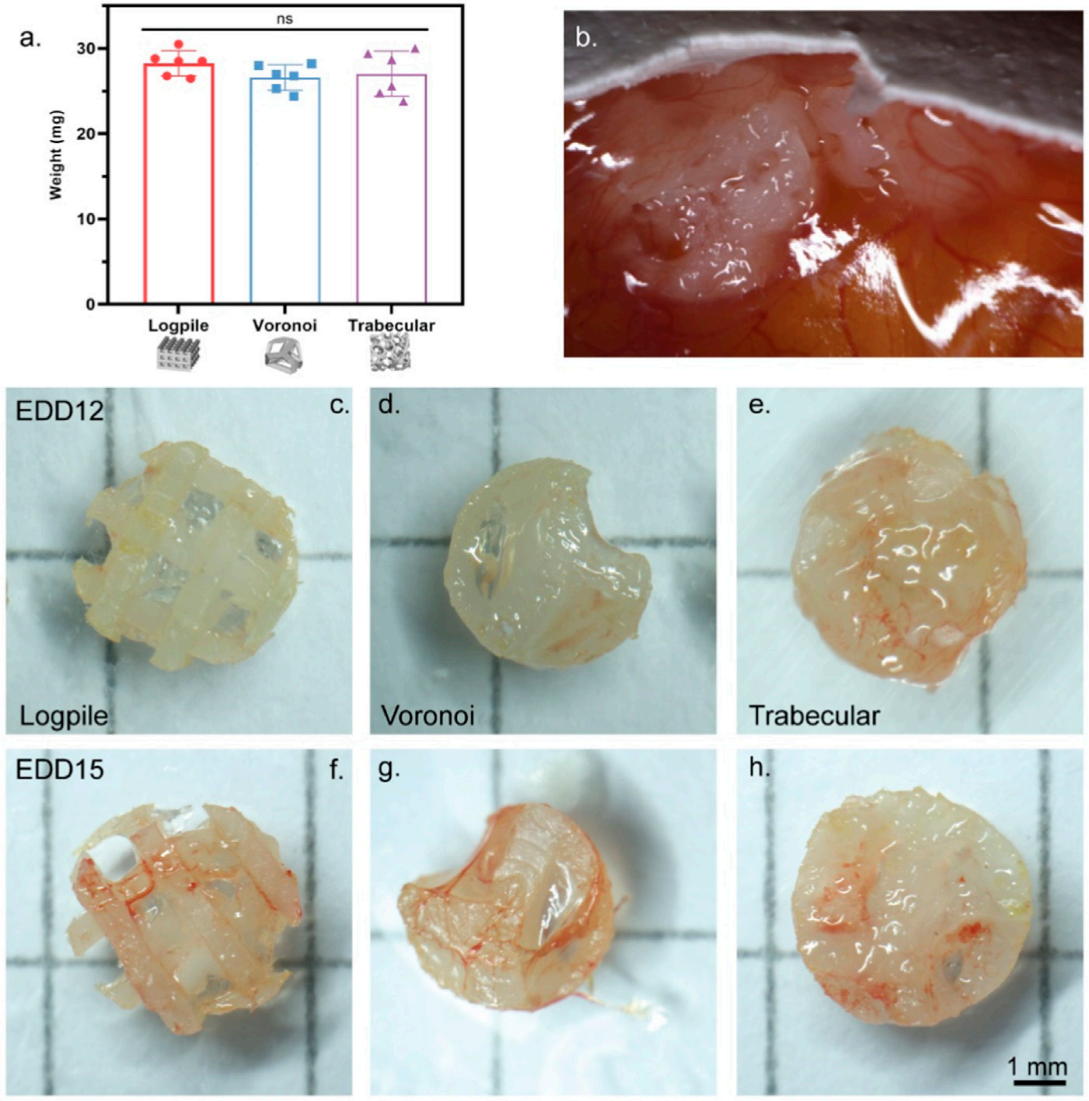
CAM model setup. **(A)** Weight of NEST PCL scaffold plus GelMA. Error bar represents standard deviation, n = 6. **(B)** Representative high magnification of a macroscopic image showing hydrogel scaffolds on EDD 12 fully or partially embedded in the CAM. **(C–H)** Representative macroscopic images of scaffolds explanted on EDD 12 **(C–E)** or EDD 15 **(F–H)**. Scale bar refers to all scaffolds **(C–H)**.

The porosity (65%–68%) and, therefore, volume fraction of each scaffold design were approximately matched, and the same volume of GelMA was included with each design. Therefore, each scaffold design had approximately the same weight, 28.3 ± 1.3 mg for logpile, 26.6 ± 1.4 mg for Voronoi, and 27.1 ± 2.4 mg for trabecular bone (Figure 3A). The literature shows up to six scaffolds can be placed on the CAM (seeded on EDD 9) (Kohli et al., 2020). However, in this study, a more conservative approach was taken, and only up to three hydrogel scaffolds per egg were tested. The hydrogel scaffolds were seeded on the CAM on EDD 8 and then observed until EDD 12. During these 4 days, most hydrogel scaffolds had moved to the edge of the shell where the CAM attached to the shell. By EDD 12, most of the hydrogel scaffolds were partially or fully embedded in the CAM (Figure 3B), and there was no indication of damaged vessels. Of the hydrogel scaffolds embedded in the CAM, it is unclear whether they partially sunk into the CAM or whether a layer of the CAM simply grew over the hydrogel scaffold (Figure 3B). Overall, it could be concluded that up to three hydrogel scaffolds could be seeded on the CAM without damaging the network.

The second stage in setting up the CAM model was to investigate the influence of time on the interaction of the CAM with the scaffolds. In this case, the same three designs were utilized and harvested on EDD 12 or 15. Overall, regardless of the scaffold design, from the macroscopic evaluation, an increased level of vessel infiltration from EDD 12 to 15 can be recognized (Figures 3C–H).

### 3.3 Biomaterial and scaffold design influences CAM and vessel infiltration

The literature indicates that pure PCL or GelMA allow limited vessel growth into these materials (Keshaw et al., 2010; Rameshbabu et al., 2018; Augustine et al., 2019; Cidonio et al., 2019; Rizwan et al., 2019; Aldemir Dikici et al., 2020; Kohli et al., 2020; Zehra et al., 2020; Dikici et al., 2021; Ren et al., 2021; Reys et al., 2021). To explore this, the scaffold conditions tested included the three PCL designs (logpile, Voronoi, and trabecular bone), with or without GelMA hydrogel and a GelMA-only condition. The PCL scaffolds are completely interconnected with approximately the same porosity, irrespective of the design, thereby allowing for the observed results to be attributed to the biomaterial and/or the scaffold design.

**FIGURE 4:**
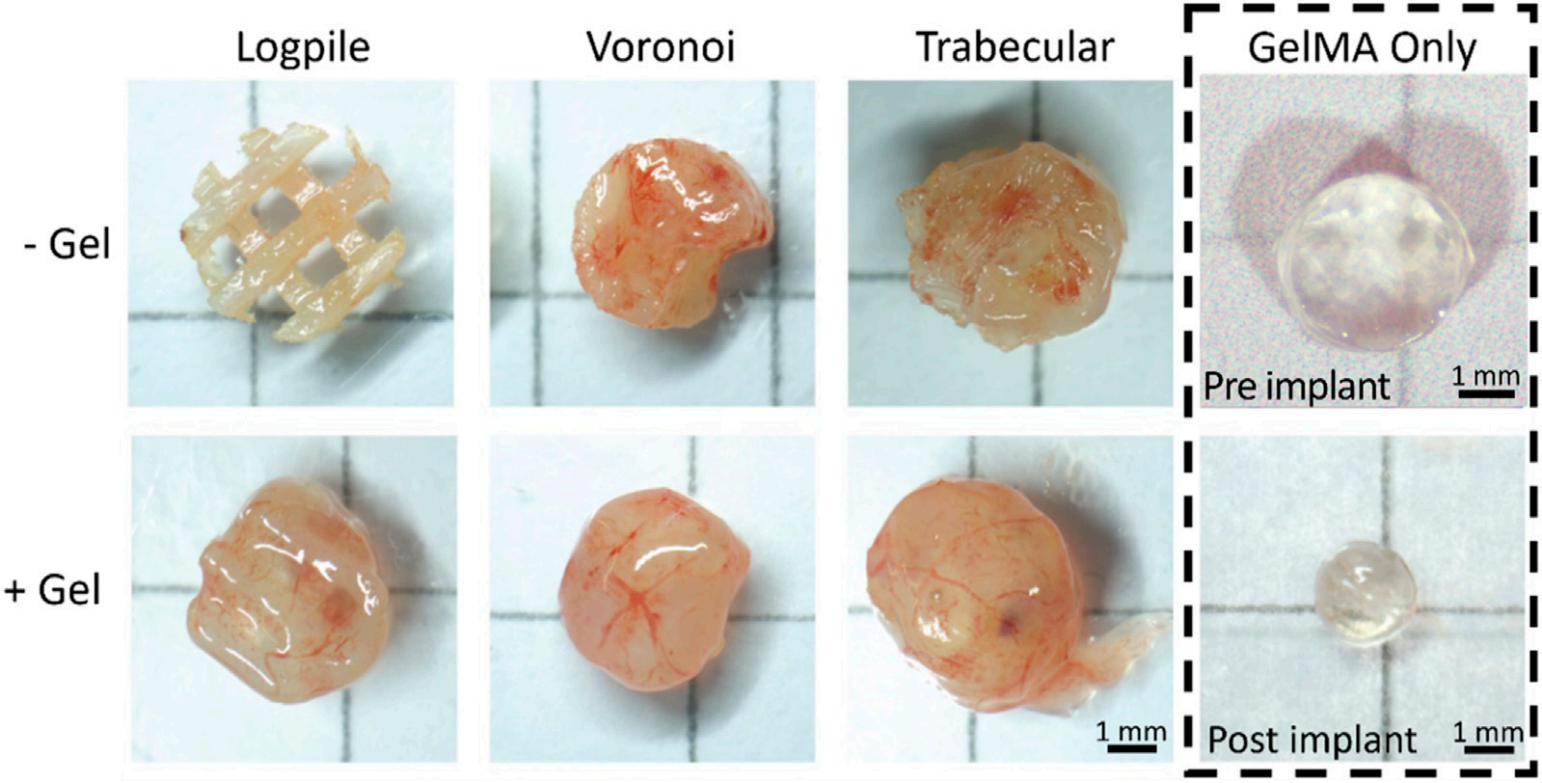
CAM assay results. Representative macroscopic images for each condition after 7 days of culture on the CAM (EDD 15). Scale bar is applicable to all hydrogel scaffolds.

After 7 days of culture on the CAM (EDD 15), all scaffolds were harvested (Figure 4). From the macroscopic images, all scaffold-only or hydrogel scaffolds showed some level of vessels infiltrated. The logpile structure without gel had the lowest level of vessel infiltration, with some blood vessels being present and little indication of new tissue infiltration. The remaining scaffold-only samples, or hydrogel scaffolds, appeared to be greatly filled with both new tissue and blood vessels, indicating that neither the PCL nor GelMA actively inhibits the infiltration of blood vessels. The GelMA-only scaffolds at the time of explant showed a reduced size, and while some limited vessels are seen macroscopically, it is unclear if they are on top or penetrated within the gel. A limitation of macroscopic imaging is the difficulty in distinguishing whether the vessels infiltrated through the scaffold or simply surrounded it (Rameshbabu et al., 2018; Augustine et al., 2019; Rizwan et al., 2019).

**FIGURE 5:**
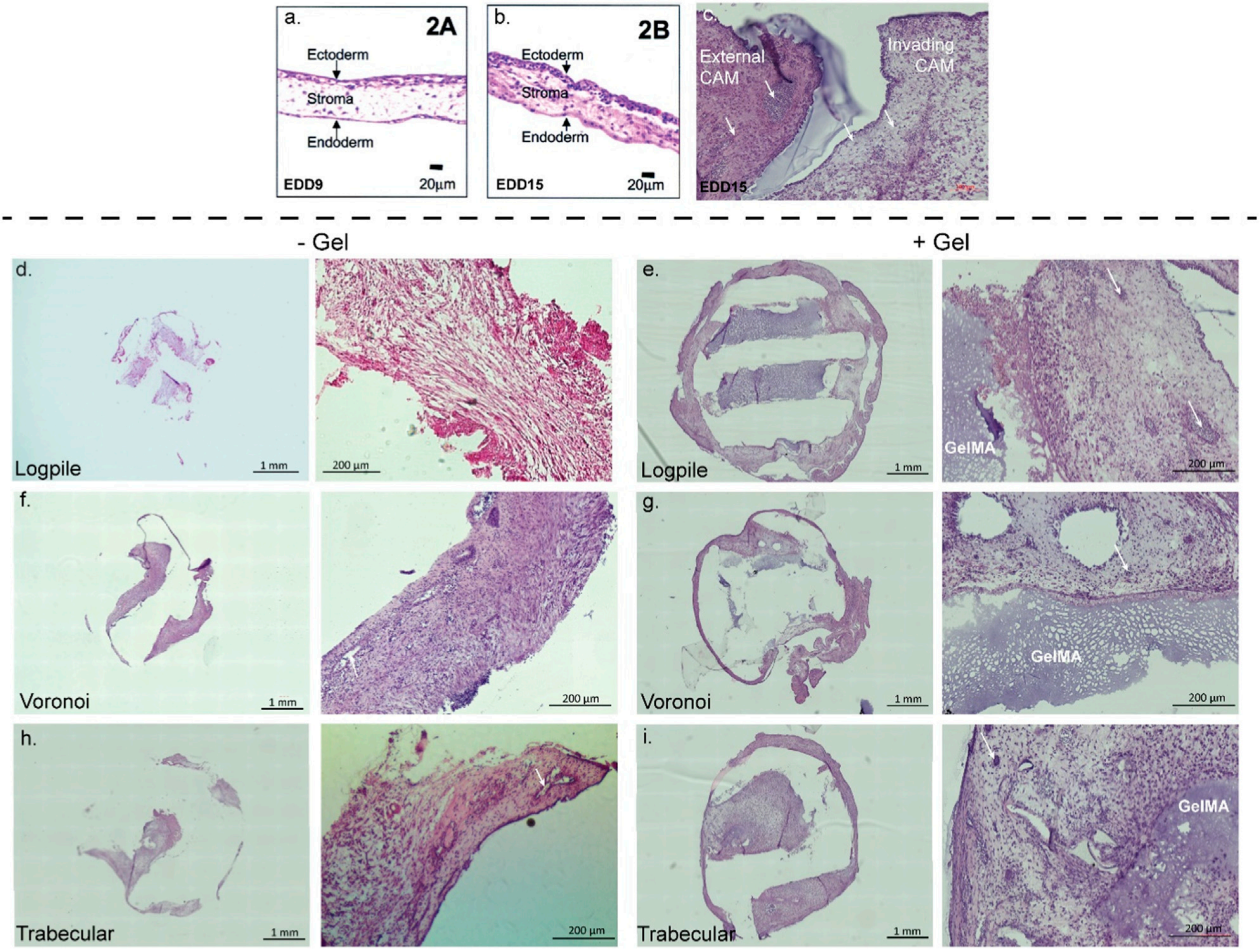
CAM assay results: histological staining. **(A,B)** CAM development from the literature. Figures from Valdes et al. (2002). **(C)** Representative image of 7-µm cryosections from one hydrogel scaffold stained with H&E to highlight the CAM surrounding the scaffold (external CAM) and CAM tissue within the scaffold (invading CAM). **(D–I)** Representative image of 7-µm cryosections stained with H&E from the different design structures explanted at EDD 15, in the absence (-gel) or presence (+gel) of GelMA hydrogel. GelMA is labeled, and white arrows show blood vessels (not all GelMA and vessels labeled).

The CAM tissue is composed of three distinctive layers (Figures 5A, B). The ectoderm or upper chorionic epithelium is the layer of the CAM close to the shell and features a multi-layer epithelium (Valdes et al., 2002; Nowak-Sliwinska et al., 2014; Yuan et al., 2014). The middle layer (stroma or mesoderm) contains connective tissue and blood vessels, and the endoderm or lower allantoic epithelium features a single-layer epithelium (Valdes et al., 2002; Nowak-Sliwinska et al., 2014; Yuan et al., 2014). Valdes et al. (2002) showed that at EDD 9, the stroma is loosely connected (Figure 5A), while by EDD 15, it is denser with more cells and connective tissue present (Figure 5B) (Valdes et al., 2002). Figure 5C shows a representative section from our hydrogel scaffold where there is CAM surrounding the scaffold (external CAM) and potentially CAM tissue within the scaffold (invading CAM). Taking only the density of the CAM into account, the external CAM is completely full of tissues and cells, while the potentially invading CAM is sparser and, therefore, can be considered newer and less developed. Based on this, the composition of the tissue inside the scaffolds is likely to be invading CAM of variable densities.

In all scaffolds containing conditions, the PCL was physically dislodged during sectioning and, instead, is shown as an empty space in Figure 5. In the hydrogel scaffold groups, it appears the new CAM is primarily formed where the GelMA is not present (Figures 5E, G, I). Since no infiltration of chicken endothelial cells or blood vessels into the GelMA construct was observed (Supplementary Figure S2), the hypothesis is that GelMA degrades, and the new invading CAM and blood vessels could take over the space. In the PCL-only conditions (no GelMA), the new tissue takes over all the space where the PCL is not present (Figures 5D, F, H). As new CAM and blood vessels were present in each of the three designs but not in GelMA only, it suggests that the biomaterial composition influences the level of infiltration more than the design of the scaffold.

**FIGURE 6:**
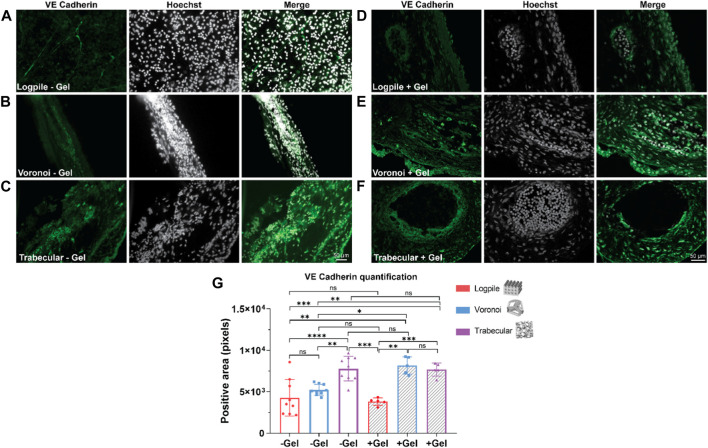
CAM assay results: immunofluorescent staining to assess vessel infiltration. **(A–F)** Representative images of 7-µm cryosections stained from the different design’s structures explanted at EDD 15, immunostained with VE cadherin (green signal) and counterstained with Hoechst to detect cell nuclei (white signal). Scale bar in **(C)** is applied to **(A–C)**, and scale bar in **(F)** is applied to **(D–F)**. **(G)** Bar graph represents the quantification of the positive area for VE cadherin calculated for the indicated groups. * is *p* ≤ 0.05; ** is *p* ≤ 0.01; *** is *p* ≤ 0.001; and **** is *p* ≤ 0.0001.

To determine the degree of blood vessel infiltration, we assessed VE cadherin expression in the three scaffold models. VE cadherin was chosen as it plays an important role in controlling the cohesion and organization of intercellular junctions in endothelial cells (Vestweber, 2008; Rho et al., 2017). Figure 6 shows representative images of the expression of VE cadherin of the three scaffold designs with and without GelMA. All three scaffold models are strongly cellularized as assessed by the nuclear labeling (Hoechst staining in white, Figure 6), and all scaffolds also show varying degrees of vessel infiltration (VE cadherin staining in green, Figure 6). The logpile design (Figures 6A, D) displays limited vessel infiltration in the absence of hydrogel, while in the presence of GelMA, vessels with thin walls and low signal intensity are detectable. The Voronoi design (Figures 6B, E) shows a greater number of vessels with thicker walls and more intense VE cadherin expression both in the presence and absence of GelMA. Finally, the trabecular design without GelMA (Figure 6C) shows an overall greater intensity of staining compared to the logpile and Voronoi, and in the presence of GelMA, large blood vessels with a thick endothelial wall are detectable. The quantification shows significantly more VE cadherin expression in the Voronoi and trabecular design than in the logpile, regardless of the inclusion of GelMA (Figure 6G).

### 3.4 Biomaterial and scaffold design influences osteogenic differentiation

After evaluating blood vessel infiltration, the degree of osteogenic differentiation of the tissues grown in the three scaffolds was also determined through the expression of collagen type I, osteocalcin, and calcium mineralization. The two markers were specifically selected to detect the early-stage osteogenic differentiation with collagen type I, while osteocalcin, known as bone GLA protein, was selected to detect late osteogenesis (Tsao et al., 2017; Salhotra et al., 2020).

**FIGURE 7:**
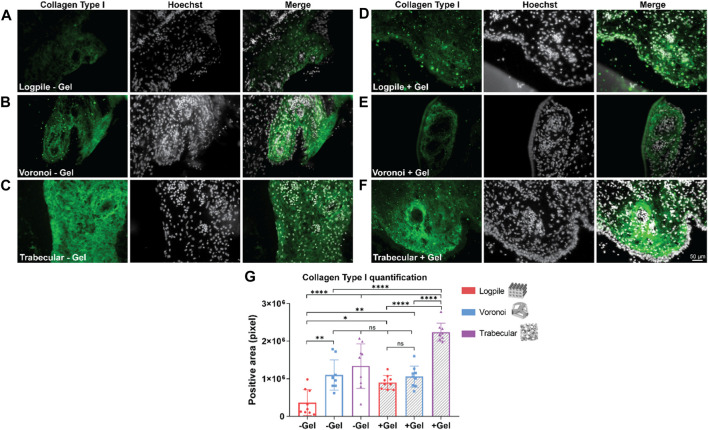
CAM assay results: immunofluorescent staining to assess osteogenic differentiation. **(A–F)** Representative images of 7-µm cryosections stained from the different design’s structures explanted at EDD 15, immunostained with collagen type I (green signal) and counterstained with Hoechst to detect cell nuclei (white signal). Scale bar in **(F)** is applied to **(A–F)**. **(G)** Bar graph represents the quantification of the positive areas for collagen type I calculated for the indicated groups. * is *p* ≤ 0.05; ** is *p* ≤ 0.01; *** is *p* ≤ 0.001; and **** is *p* ≤ 0.0001.

The staining analysis shows that the logpile design in the absence of GelMA (Figure 7A) displays a lower intensity of collagen type I compared to the condition where GelMA is present (Figure 7D). The Voronoi designs show a similar intensity of collagen type I expression with or without GelMA (Figures 7B, E). The trabecular design without GelMA (Figure 7C) shows the most homogenous and intense staining compared to the other two groups (Figure 7F). The quantification of the area of positive collagen type I staining (Figure 7G) showed the trabecular design again had a significantly greater area covered compared to the logpile and Voronoi designs, especially in the presence of GelMA.

**FIGURE 8:**
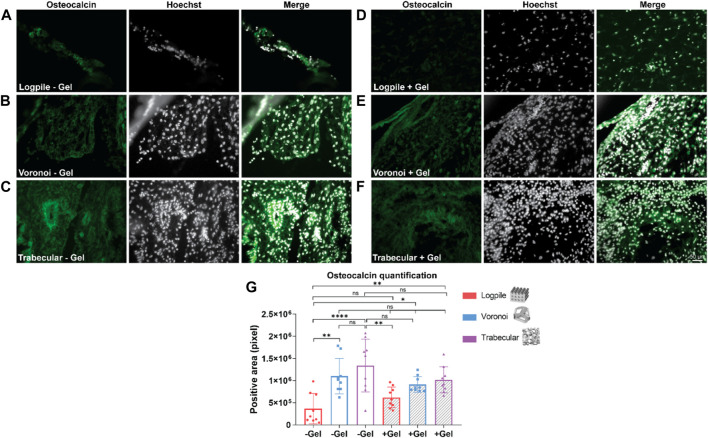
CAM assay results: immunofluorescent staining to assess osteogenic differentiation. **(A–F)** Representative images of 7-µm cryosections stained from the different design’s structures explanted at EDD 15, immunostained with osteocalcin (green signal) and counterstained with Hoechst to detect cell nuclei (white signal). Scale bar in **(F)** is applied to **(A–F)**. **(G)** Bar graph represents the quantification of the positive areas for osteocalcin calculated for the indicated groups. * is *p* ≤ 0.05; ** is *p* ≤ 0.01; *** is *p* ≤ 0.001; and **** is *p* ≤ 0.0001.

The staining analysis performed using osteocalcin as a marker of osteogenesis revealed that the logpile design, regardless of the inclusion of GelMA (Figures 8A, D), shows a weak expression of osteocalcin. The Voronoi design shows greater expression compared to the logpile and a similar intensity and distribution in both the absence (Figure 8B) and presence (Figure 8E) of GelMA. Finally, the trabecular design shows a similar distribution compared to the Voronoi; however, without GelMA (Figure 8C), there are more intense regions of expression which appear to surround the perimeter of the blood vessels. This is not seen when GelMA is included within the PCL scaffold (Figure 8F). The quantification of the positive areas shows a significant increase in the Voronoi and trabecular designs, particularly in the absence of GelMA (Figure 8G).

**FIGURE 9:**
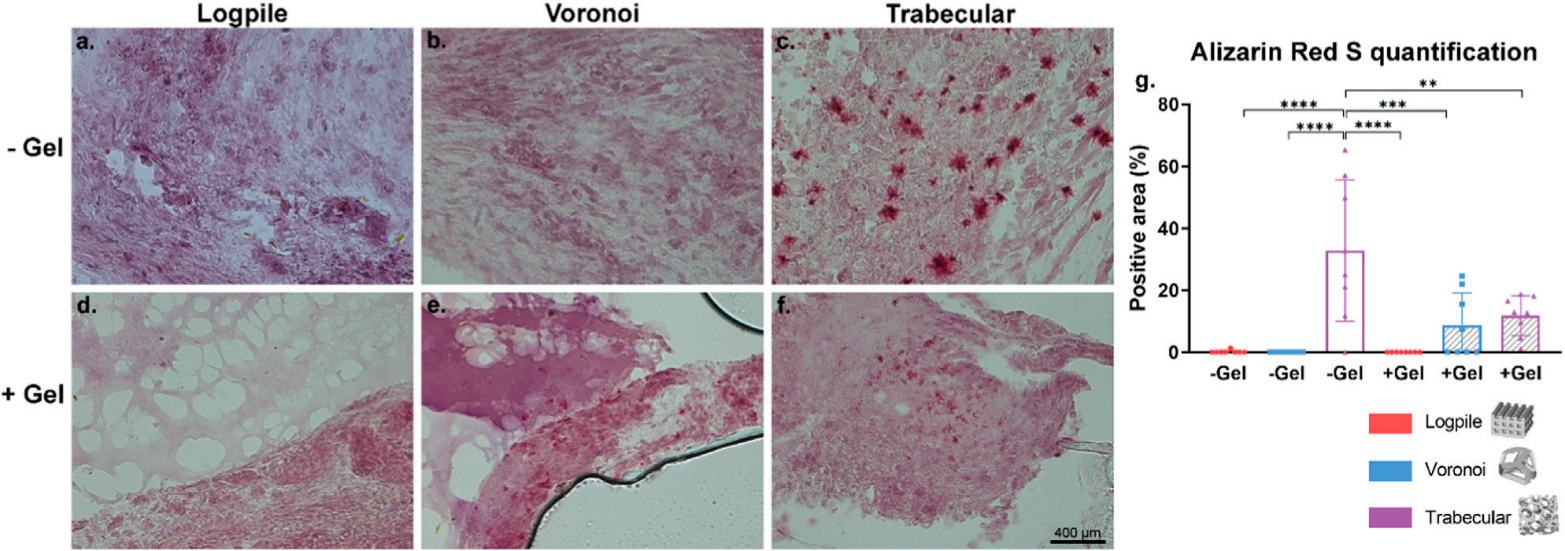
CAM assay results: Alizarin Red S staining to assess calcium mineralization. **(A–F)** Representative images of 7-µm cryosections stained from the different design’s structures explanted at EDD 15, stained with Alizarin Red S. Pink color indicates background staining of the GelMA and CAM. Deep red coloring indicates a true signal for calcium deposit. Scale bar in **(F)** is applied to **(A–F)**. **(G)** Bar graph represents the quantification of the positive areas for Alizarin Red S calculated for the indicated groups. Where no stars are shown, the relationship is not significant. * is *p* ≤ 0.05; ** is *p* ≤ 0.01; *** is *p* ≤ 0.001; and **** is *p* ≤ 0.0001.

The mineralization process is essential for bone regeneration together with the infiltration of blood vessels. Alizarin Red S stain was used to identify the calcium in the tissue and is shown in the form of red granules. Of the six conditions tested, the logpile design displayed no detectable calcium within the scaffolds (Figures 9A, B). In the Voronoi scaffolds, in the presence of hydrogel, there were some very limited indications of calcium deposits (Figure 9D), while without the hydrogel, no calcium deposits were observed (Figure 9C). The trabecular design without hydrogel (Figure 9E) had the strongest signal of Alizarin Red S with a reasonably homogenous distribution of calcium deposits across the scaffold. The trabecular design with the hydrogel (Figure 9F) had a weak indication of calcium deposits, similar to that of Voronoi with hydrogel. It is unclear whether the calcium is free calcium from the eggshell that has been able to diffuse within the scaffolds or if it has been produced by any of the chicken cells. The images in Figure 9 were captured with a ×40 objective, therefore showing that the calcium deposits are small and suggesting that after 7 days on the CAM, the mineralization of the matrix is only just beginning. Nonetheless, from the Alizarin Red S staining and quantification (Figure 9G), the trabecular design is the most favorable for producing a mineralized matrix, which is crucial for bone regeneration.

## 4 Conclusion

The creation of a 3D scaffold for the repair of bone needs to consider the mechanical reinforcement of the overall structure along with the ability to allow vascularization of the structure. The different designs of scaffolds fabricated by NEST3D printing were shown to influence their stiffness. The human trabecular bone design with a stiffness of 25.93 ± 4.16 MPa better resembled the native bone than the standard 90° logpile pattern with a stiffness of 14.68 ± 5.36 MPa.

Despite the influence of a design on stiffness, the chick CAM tissue was able to invade all conditions that involved a PCL scaffold, regardless of the scaffold design or the presence of GelMA. Therefore, this study showed that there is no benefit, in terms of CAM and vessel infiltration, of adding pure GelMA. The advantage lies in including GelMA as a carrier for additional components such as cells, growth factors, or ceramic particles to enhance osteogenic differentiation. Of note, the CAM naturally rapidly expands; therefore, it would need to be further investigated whether the vessel infiltration results would compare in an *in vivo* bone defect, just at a different rate. While PCL and GelMA composite scaffolds are regularly used for *in vitro* studies, currently, there is no literature in which this composite scaffold has been placed on the CAM. The existing literature on PCL only or GelMA only showed that both polymers have limited ability allowing for vascularization infiltration (Cidonio et al., 2019; Kohli et al., 2020). Our results instead show that PCL-only or PCL and GelMA composites allow complete CAM infiltration. Overall, the trabecular design was consistently the best design for vascularization and osteogenic differentiation as detected via staining for collagen type I, osteocalcin, and calcium deposit quantification. However, especially for the mineralization assessment, a longer experiment would be required given the small quantities present after the 7 days on the CAM. The capacity of inducing osteogenesis from the CAM may be due to the presence of mesenchymal cells with inherited capacity to differentiate into osteoblasts. The CAM forms during chicken development starting from EDD 5 by the fusion of the allantois and the chorion membrane, which are separated by a mesenchymal layer (Makanya et al., 2016; Halgrain et al., 2022a). While the external layers mainly consist of epithelial cells, the mesenchymal layer comprises fibroblast-like cells that assume a cuboidal form during maturation (Gabrielli and Accili, 2010). Adult mesenchymal stem cells (MSCs) in humans and animal species are osteo-competent cells and adopt a cuboidal form when differentiating toward the osteogenic phenotype. Hence, although direct evidence is lacking, it is plausible that these fibroblast-like cuboidal cells in the CAM are MSCs. Furthermore, during the entire embryonic incubation period from EDD 5 to 20, the mesoderm layer is the largest component and is penetrated by small and large vessels (Ribatti et al., 2021). MSCs are detected in vascularized tissues, and MSCs are even hypothesized to have a perivascular origin (Crisan et al., 2008); therefore, it is feasible that the CAM in physiological conditions contains MSCs, and, thus, osteogenic cells. The chicken eggshell is a highly organized structure composed of 95% calcium carbonate. Under physiological conditions, the CAM is a dynamic organ whose function is to regulate gas exchange, pH levels, water, and mineral content (via calcium and magnesium solubilization and transfer from the eggshell to the embryo). Starting from EDD 11, the eggshell undergoes significant changes accompanied by a gradual thinning of the shell, and calcium carbonate is gradually resorbed from the shell to contribute to the mineralization of the embryo’s skeleton (Halgrain et al., 2022a; Halgrain et al., 2022b; Biesek, 2023). There are different types of cells in the chorionic epithelium during the various stages of calcium resorption, and one of them could be responsible for mineralization (Halgrain et al., 2022a).

The ability of the PCL and GelMA composite scaffolds to support complete CAM infiltration suggests its promise as a candidate for further investigation in tissue engineering and regenerative medicine. Additionally, the insights into the scaffold design’s impact on tissue outcomes provide valuable guidance for designing effective biomimetic structures that can aid in tissue repair and regeneration. Further research in this direction could potentially lead to innovative approaches for addressing tissue loss and advancing clinical treatments.

## 5 Translational impact statement

Bone repair starts with the formation of a vascular network which promotes tissue remodeling and leads to the formation of mineralized tissue. NEST3D printing offers a versatile method for creating scaffolds with varied geometries, and this study reveals their influence on mechanical properties and vascularization. Assessing our findings with a CAM assay, we found that the trabecular bone motif showed superior mineralized matrix production, highlighting the importance of scaffold design in bone regeneration. These insights offer valuable guidance for optimizing tissue-engineered implants, emphasizing the critical interplay between design parameters and biomaterial selection in fostering successful bone regeneration outcomes.

## Data Availability

The original contributions presented in the study are included in the article/Supplementary Material; further inquiries can be directed to the corresponding author.
