# Supplementary material for: NEST3D printed bone-mimicking scaffolds: assessment of the effect of geometrical design on stiffness and angiogenic potential
Source: Front Cell Dev Biol. 2024 Mar 7;12:1353154. doi: 10.3389/fcell.2024.1353154 (PMC10955058; doi:10.3389/fcell.2024.1353154)
Supplement: Supplementary file 1 [file DataSheet1.docx]

**Supplementary information: NEST3D printed bone mimicking scaffolds: Assessment of geometrical design on stiffness and angiogenic potential**

Stephanie E. Doyle*, Micaela Pannella*, Carmine Onofrillo, Chiara Bellotti, Claudia Di Bella, Cathal D. O’Connell, Elena Pirogova, Enrico Lucarelli*, Serena Duchi*

*These authors contributed equally


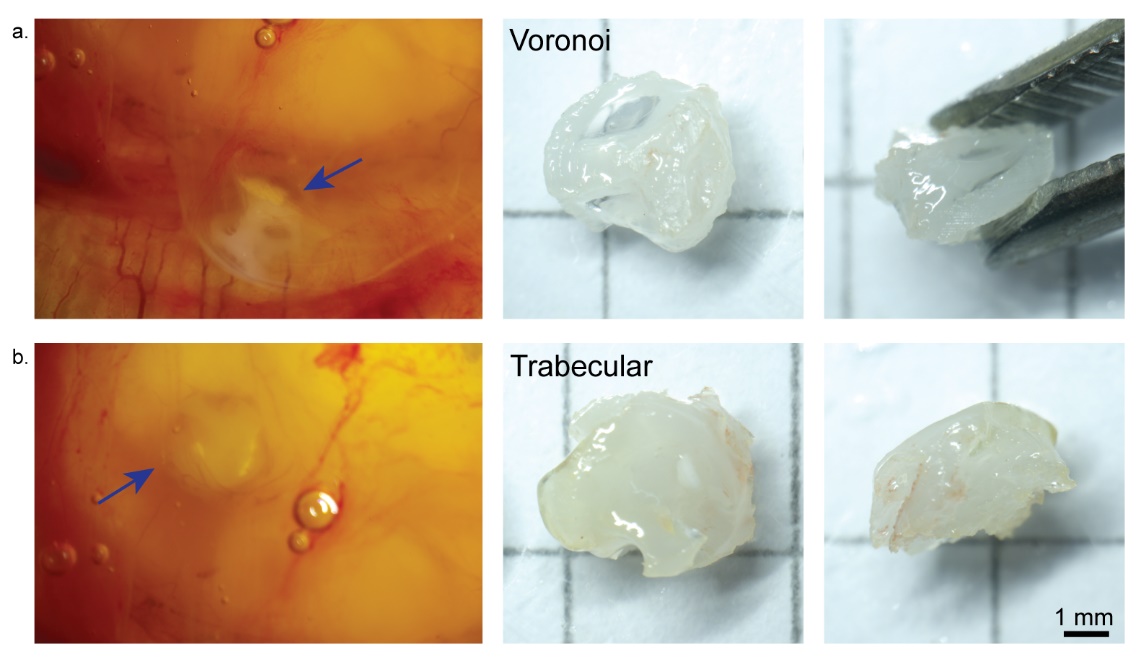


**Supplementary Figure 1.** CAM model set-up: troubleshooting. Sunken scaffolds when harvested on EDD15. (**a**) A Voronoi scaffold which was not found on the surface of the CAM and instead appears to have ended up in the amniotic sac. (**b**) A trabecular scaffold which also sunk through the CAM and may have ended up in the yolk. Both scaffolds when harvested had little to no blood vessels present.


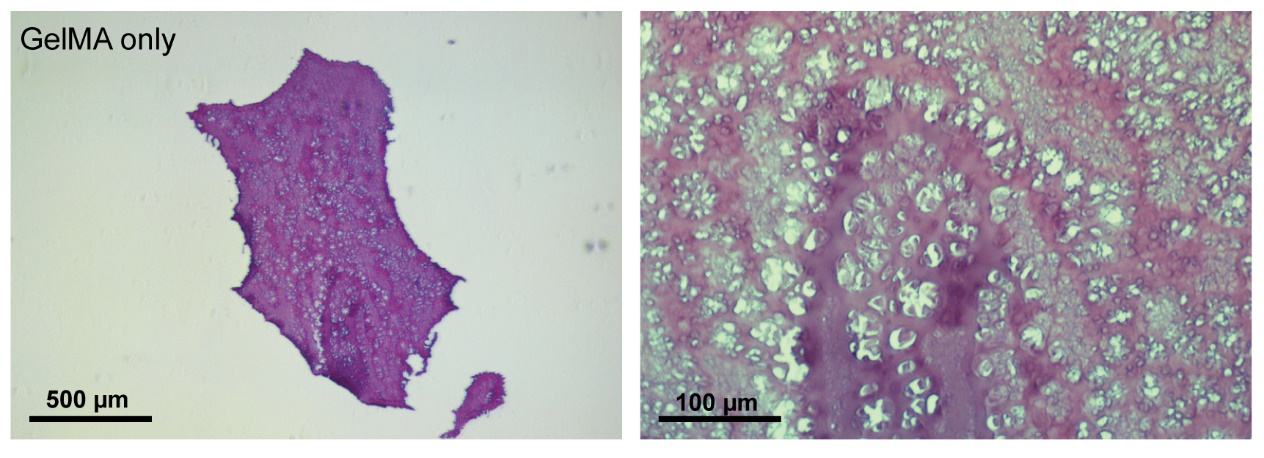


**Supplementary Figure 2.** H&E staining on 7 µm cyrosections of GelMA only hydrogel.
